# Reporting of Results from Animal Studies

**DOI:** 10.1289/ehp.1307676

**Published:** 2013-12-01

**Authors:** Hugh A. Tilson, Jane C. Schroeder

**Affiliations:** 1 Editor-in-Chief**,***Environmental Health Perspectives***,** tilsonha@niehs.nih.gov; 2 Science Editor, *Environmental Health Perspectives*, schroederjc@niehs.nih.gov

One of the key elements of the scientific process is reproducibility: Experimental results generated in one laboratory must be reproducible by others working independently. Therefore, it is of concern to us as editors of a major scientific journal to learn that only 6 of 53 “landmark” preclinical cancer studies could be reproduced (Begley and Ellis 2012). In addition, Prinz et al. (2011) reported that almost two-thirds of 67 in-house preclinical cancer projects did not replicate data previously published by others. These observations are based on preclinical cancer studies, and it is not known how pervasive this problem may be in other disciplines, including environmental health research.

Vasilevsky et al. (2013) pointed out that the reproducibility of scientific research depends in large measure on whether the “materials and methods” of a paper are described such that other investigators are able to independently replicate effects observed in the original study. Unfortunately, the degree to which sufficient methodological details are provided can be problematic. For example, Kilkenny et al. (2009) published the results of a systematic review of original *in vivo* research involving laboratory animals. Of the 271 papers they evaluated, only 60% provided information about the number of animals used or described in detail the species, strain, sex, age, or weight of the animals. The authors also noted that about 30% of the papers lacked sufficient details concerning the statistical analyses. Findings such as these indicate that the lack of methodological detail could be a major barrier to subsequent attempts to replicate the work of others.

According to Ransohoff and Gourlay (2010), “any study’s reliability is determined by [the] investigators’ choices about critical details of research design and conduct.” Some choices could lead to bias or the “systematic erroneous association of some characteristic with a group in a way that distorts a comparison with another group” (Ransohoff 2005). Key approaches to reduce bias include the randomization of groups and blinding (Krauth et al. 2013). However, randomization and blinding are not used universally in preclinical research. Van der Worp et al. (2005) reported that fewer than half of the 45 preclinical studies they reviewed included randomization of treatment, blinded administration of treatment, and blinded outcome evaluation. In addition, Kilkenny et al. (2009) found that most papers they surveyed did not use randomization or blinding.

Addressing concerns about the reproducibility of research findings will require cooperation and collaboration from major segments of the scientific community. From our perspective, it seems clear that many authors are not sufficiently trained in experimental design or the importance of controlling for sources of bias in their studies. We believe that courses on the principles of experimental design should be included at the graduate level. Many academic departments and governmental institutions now require students and employees to take courses on ethics. We believe that it is equally important to train students and young investigators about experimental design and how to be transparent in reporting their results in the peer-reviewed literature. In addition, we believe that it is important for study sections and funding agencies to critically evaluate experimental designs described in grant proposals under consideration. Finally, journals can be more proactive by insisting that critical methodological details be included in papers submitted for possible publication. Associate editors and reviewers need to be instructed to consider experimental design as part of the peer-review process.

Kilkenny et al. (2010) suggested that the scientific community would benefit from guidance about the information needed in a research article. As an example, they describe the CONSORT Statement for randomized clinical trials (Moher et al. 2001). Many journals have endorsed the CONSORT guidelines, and there is some evidence that use of these guidelines has improved the quality of papers on clinical trials (Kane et al. 2007).

In June 2009, an expert working group consisting of researchers, statisticians, and journal editors met to develop a checklist that could be used improve the reporting of research using animals (Kilkenny et al. 2010). The product of this effort is the Animal Research: Reporting of *In Vivo* Experiments (ARRIVE) guidelines (http://www.nc3rs.org.uk/ARRIVE). These guidelines consist of 20 items identifying minimum information required for scientific research reporting results from animal studies. According to Kilkenny et al. (2010), the guidelines were developed “to maximise the output from research using animals by optimising the information that is provided in publications on the design, conduct, and analysis of the experiments.”

With this editorial, *EHP* joins the growing list of journals that endorse the ARRIVE guidelines for animal research. We encourage authors to review these guidelines when designing their studies and to use them in writing papers for submission to *EHP*. We encourage our Associate Editors and peer reviewers to keep in mind the principles articulated in the ARRIVE guidelines when evaluating papers involving animal research. We believe that by adhering to the guidelines, the quality of the papers will improve and the potential for reproducibility of findings will increase.

The ARRIVE guidelines focus on *in vivo* animal research; other types of research not covered by the ARRIVE guidelines also need to be considered. There is a need for clear and complete descriptions of methods across all disciplines.
